# Bed Particle Layer Formation and Characteristics of Ilmenite Bed Particles Utilized in Fluidized Bed Combustion of Chicken Litter

**DOI:** 10.1021/acsomega.5c01057

**Published:** 2025-06-12

**Authors:** Ali Valizadeh, Thomas Karl Hannl, Juraj Priščák, Matthias Kuba, Marcus Öhman

**Affiliations:** † Energy Engineering, Division of Energy Science, 407846 Luleå University of Technology, Luleå SE-971 87, Sweden; ‡ 210802 BEST-Bioenergy and Sustainable Technologies GmbH, Inffeldgasse 21b, Graz A-8010, Austria; § Institute of Chemical and Energy Engineering (IVET), BOKU University, Muthgasse 107/I, Vienna A-1190, Austria

## Abstract

Despite extensive research on the bed particle layer formation and characteristics of ilmenite bed particles in fluidized bed combustion and gasification of woody biomass (characterized by high Ca and K content), a significant knowledge gap exists when P-rich fuels are used. Chicken litter, as a P-rich biomass, presents a promising alternative biomass for energy conversion processes. Therefore, this study aims to investigate the bed particle layer formation and characteristics of ilmenite bed particles during fluidized bed combustion of chicken litter. The bed particle layer formation process and characteristics for ilmenite bed particles utilized in a 5 kW_th_ bubbling fluidized bed combustion of chicken litter were studied in this work. Bed samples were taken after 4, 8, 12, and 16 h from the start-up and were analyzed via SEM/EDS. The findings highlighted that the initial stage of bed particle layer formation is similar to what was observed in fluidized bed combustion of woody biomass and is driven by the reaction of fuel-derived Ca-rich particles with the bed particle, leading to the formation of the inner layer. As time progresses, the deposition of fuel-derived bed ash, containing mainly Ca, P, Mg, and K, forms an outer layer that uniformly covers the entire bed particle surface, irrespective of the bed particle’s surface morphologies. Additionally, the outward migration of Fe from ilmenite bed particles is significantly constrained when utilizing chicken litter, a Ca- and P-rich fuel, due to the formation of a bed particle layer that uniformly covers the bed particle surface.

## Graphical abstract

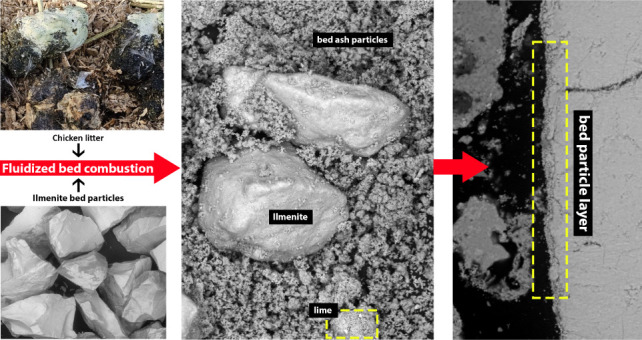

## 1 Introduction

Climate change, driven primarily by the emission of greenhouse gases from the combustion of fossil fuels, represents one of the most significant challenges of our time.^1,2^ The adverse effects of global warming, such as rising sea levels, increased frequency of extreme weather events, and disruptions to ecosystems, underscore the urgent need to transition to more sustainable energy sources.^3–5^ Among the various alternatives to fossil fuels, biomass is a promising option.^6^ Biomass fuels, derived from organic materials such as agricultural residues and forestry byproducts, offer a renewable energy source. One particularly notable type of biomass fuel is biogenic waste derived from livestock breeding, such as chicken litter (CL).^7,8^ Utilizing CL as a fuel in energy conversion processes (e.g., combustion) provides an effective solution for managing this abundant waste and contributes to reducing greenhouse gas emissions.^9^

However, despite their environmental benefits, the use of biogenic wastes such as agricultural residues, sludges, manures, and litter presents certain challenges. These include higher heterogeneity, moisture content, ash content, and lower heating value compared to conventional fossil fuels.^10^ These types of biomass fuels are often characterized by high K and P/Si content.^11^ Additionally, Ca is another abundant element in CL.^7^ Ca is crucial for chickens, especially laying hens, for strong bones and eggshell formation. Given that the average Ca to P ratio (≈1:8.6) in typical cereals is lower than the optimal level (2:1), additional Ca supplementation is necessary.^12^ Therefore, to meet their Ca needs, chickens are provided with Ca-rich feed (e.g., limestone or oyster shells).^13^ Excess Ca that is not absorbed during the chicken’s digestion process will be excreted in their manure, contributing to the Ca content in CL.^14,15^ Consequently, the majority of the Ca observed in CL originates from the supplemental Ca sources.

Among the various technologies available for energy conversion of biomass fuels, fluidized bed combustion stands out as a particularly advantageous option. Its versatility in handling a wide range of fuel mixtures, operating at moderate temperatures (<1000 °C), and enhanced fuel burnout contribute to its status as a preferred choice for energy conversion from biomass fuels.^10,16–19^

Ilmenite has been extensively studied in the context of fluidized bed, especially in chemical looping combustion (CLC), considering its ability to transport oxygen throughout the bed and provide more uniform combustion conditions.^20–26^ The oxygen-carrying ability of ilmenite bed particles is associated with the migration of Fe from the core to the surface of the bed particles.^27^ Under oxidized conditions, Fe can exist as pseudobrookite (Fe_2_TiO_5_) or hematite (Fe_2_O_3_), whereas under reduced conditions, it can be found as ilmenite (FeTiO_3_) or magnetite (Fe_3_O_4_).^28^ However, the outward migration of Fe creates porous regions within the bed particles’ core, which eventually compromises their structural integrity and mechanical stability.^27,29^

The performance of ilmenite bed particles in the process is significantly influenced by the formation of the bed particle layer.^20,29,30^ The layer formation, which is a well-documented phenomenon in fluidized bed processes, arises from the interaction between the bed material and the ash-forming matter in the biomass fuel.^31–36^ There are both challenges and opportunities associated with the formation of the bed particle layer. In some instances, this layer can lead to agglomeration of the bed particles.^37,38^ Bed particle fragmentation and deposition of the fine fragments in the downstream facilities (e.g., cyclones and return legs) pose further problems associated with bed particle layer formation.^39^ However, a positive effect of the bed particle layer on tar reduction has been reported in the thermal conversion of biomass in fluidized beds.^40^

The formation of the bed particle layer can occur through two distin mechanisms, the “reaction mechanism” and the “deposition mechanism”, each resulting in specific bed particle layer characteristics. In the reaction mechanism, the bed particle layer forms through the direct reaction of fuel-derived Ca- and K-rich compounds with the bed material, resulting in a Ca-silicate-rich layer on the bed particle surface. This occurs when biomass rich in Ca and K (e.g., woody biomass) is used.^31–33,41,42^ In the deposition mechanism, the bed particle layer develops through the accumulation of ash-derived low-temperature melting silicates and/or phosphates on the bed particle surface. This can occur when the biomass fuel is rich in Si, and/or P.^43–45^

Bed particle layer formation on ilmenite bed particles utilized in fluidized bed combustion of various woody biomass fuels (which are characterized by high Ca and K contents) has been extensively studied and documented in the literature.^20,27,29,30^ In these cases, bed particle layer formation begins with Ca-rich coarse ash particles (primarily present as CaO at typical combustion temperatures)^46^ adhering to the bed particle surface upon impact. Consequently, Ca in its ionic form diffuses into the bed particle. The diffused Ca then reacts with the bed material to form Ca-titanate, which is referred to as the inner layer. Simultaneously, Fe from the bed material migrates toward the bed particle surface, establishing an Fe-rich outer layer, while regions depleted of Fe become porous. This outer layer is not uniformly distributed across different surface morphologies but is mainly observed on the convex areas. In conjunction with the iron migration, fuel-derived K compounds diffuse into the bed particle core, forming K-titanate, which is predominantly found in the Fe-depleted (porous) regions. Over time, the Ca-rich inner layer thickens, while the Fe-rich outer layer disappears due to attrition between the bed particles in the fluidized bed. In aged bed particles, the thick Ca-rich inner layer formed on the bed particles surface inhibits further outward migration of Fe, resulting in the formation of an Fe-rich sublayer beneath the inner layer. However, Fe can still diffuse outward, and K can penetrate into the bed particle core through concave areas where the Ca-rich inner layer is not developed completely.^20,27^ The formation of K-titanate, while compromising the physical integrity of ilmenite, promotes iron migration to the particle surface, which improves ilmenite’s capacity to release and absorb oxygen during alternating reduction–oxidation cycles in CLC.^28,30^ However, as the Fe-rich outer layer is abraded over time and the access of Fe to the bed particle surface becomes limited, aged ilmenite bed particles exhibit a lower oxygen-carrying capacity compared to those in the early stages of the process.^20^

The dominance of Ca in bed particle layer formation when woody biomass is used is a well-understood phenomenon across various bed material types, including quartz, feldspar, olivine, and ilmenite. However, when P-rich fuels are used, studies on bed materials such as quartz and feldspar have shown a different process, dominated by the formation of low-temperature melting phosphates.^11,15,43,46–48^ In these cases, the direct adhesion of ash-derived phosphates forms the bed particle layer, resulting in characteristics that differ from those observed with Ca-rich woody fuels and consequently affecting the behavior of the bed particle in the combustion/gasification process.

Although there is a well-established understanding of bed particle layer formation and its effects on the oxygen-carrying capacity and structural integrity of ilmenite bed particles during fluidized bed combustion of woody biomass, a knowledge gap remains concerning bed particle layer formation on this bed material when fuels other than woody biomass (e.g., P-rich fuels) are used. In this case, the bed particle layer formation is expected to shift from the reaction mechanism to the deposition mechanism.^43,46^ Consequently, due to differences in bed particle layer characteristics (e.g., surface coverage and composition), the performance of ilmenite—particularly its oxygen-carrying ability and structural integrity—may be affected in the process. Therefore, understanding these effects is crucial for the commercialization of this promising bed material as a bed material for a wider range of biomass fuels. Moreover, it enhances energy security by enabling the utilization of diverse biomass types and improves the adaptability of fluidized bed processes to different locations, depending on the availability of biomass resources.

In a recent study on the interaction of different potassium salts with ilmenite bed particles, it was shown that potassium salts such as K_2_CO_3_, K_2_SO_4_, and KCl facilitate K-titanate formation, thereby enhancing the reactivity of ilmenite toward CO and H_2_.^49^ Conversely, in the presence of KH_2_PO_4_, the reactivity decreases due to the formation of molten KPO_3_, which forms a dense, nonpermeable layer around the ilmenite particles. This KPO_3_ coating acts as a barrier, inhibiting oxygen exchange and increasing the tendency for particle agglomeration, ultimately reducing the efficiency of the oxygen carrier in CLC. However, the inclusion of Ca may alter how KH_2_PO_4_ interacts with ilmenite.^49^ Therefore, the reactivity and structural integrity of ilmenite bed particles in the process can drastically vary depending on the composition of the ash in different fuels.

This study particularly focuses on CL, which is notably rich in P and Ca and has moderate levels of K. This composition suggests the possibility of both reaction and deposition mechanisms, making CL an especially valuable focus for investigation. Therefore, the objective of this study is to examine the formation process and characteristics of bed particle layers on ilmenite during fluidized bed combustion of this P-rich fuel. Findings from the study will be compared with experiments using a Ca-rich, P-lean woody biomass fuel under similar operational conditions. This will provide insight into how different fuel compositions influence the interaction between bed particles and ash-forming matter in the fuel, as well as the resulting bed particle layer formation mechanism and characteristics.

## 2 Methodology

### 2.1 Utilized Fuel

The fuel ash samples were prepared according to the standard method EN ISO 18122:2015–11 at a final temperature of 550 °C and subsequently were analyzed using XRF. The concentration of the ash-forming elements in the utilized CL fuel is presented in Table 1. The molar ratios reveal that CL is primarily dominated by Ca accompanied by high levels of P and moderate levels of K. The composition also features a notably high P/Si molar ratio. The fuel was first dried and then pelletized into 6 mm-sized pellets. This preparation helped improve handling and flow, making the fuel more suitable for controlled feeding during the experiment.

**Table 1:** Concentration of the Main Ash-Forming Elements in the Fuel Ash Given as wt % of Element Expresses as Oxides and Atomic% of Element

Ash content based on dry basis: 25.4 wt %			
Oxide	wt %	Element	atomic% (O-free)
K_2_O	10.0	K	11.9
Na_2_O	2.8	Na	5.1
CaO	43.1	Ca	43.2
MgO	7.2	Mg	10.0
P_2_O_5_	24.5	P	19.4
SiO_2_	4.6	Si	4.3
Al_2_O_3_	1.5	Al	1.7
Fe_2_O_3_	0.5	Fe	0.4
SO_3_	4.9	S	3.5
Cl	0.1	Cl	0.2
Rest	0.9	Rest	0.4

### 2.2 Ilmenite Bed Particles

Norwegian natural ore ilmenite, with an average particle diameter of 175 μm, was used as the bed material in this study. Prior to the experimental campaign, the bed particles were dried at 100 °C for 24 h, followed by calcination in air at 600 °C for 6 h. The physical properties and chemical composition of the utilized ilmenite bed particles are presented in Table 2.

**Table 2:** Properties of the Utilized Ilmenite Bed Particles, Reused from Priscak et al.^25^ under CC by 4.0 License

Physical properties		Chemical composition in wt % (after calcination)	
Average particle diameter (*d̅*_p_), μm	175	TiO_2_	44.1
Sauter mean diameter (*d̅*_sv_), μm	140	FeO	28.8
Ø	0.8	Fe_2_O_3_	17.7
Particle density (ρ_p_), kg/m^3^	3820	SiO_2_	2.0
Bed density (ρ_b_), kg/m^3^	2290	MgO	3.6
		Al_2_O_3_	0.6
		MnO	0.3
		CaO	0.3
		SO_3_	0.1
		Cr_2_O_3_	0.1

### 2.3 Experimental Campaign

Combustion experiments were conducted in a 5 kW_th_ bench-scale bubbling fluidized bed (BFB_5_) with a total height of 1.5 m consisting of two main sections: a fluidized bed section with a diameter of 136 mm and a freeboard section with a diameter of 300 mm. The bubbling regime was maintained by a constant air flow, evenly distributed through air nozzles at the bottom of the boiler. Fuel was delivered from feedstock hoppers directly into the boiler (on-bed) using screw conveyors. To maintain an inert atmosphere in the feedstock hopper, the fuel feeding system was continuously flushed with nitrogen. Temperatures in the bed, freeboard, cyclone, filter, and fuel bunker were constantly monitored with K-type thermocouples. More details on the experimental setup can be found elsewhere.^47^

The fluidized bed was loaded with 3 kg of ilmenite bed particles before the experimental campaign, which lasted for four consecutive days. Each day, the fluidized bed was preheated using two cylindrical electrical furnaces surrounding the air preheater and the bed section. Once the bed temperature reached 500 °C, CL was fed into the boiler via a screw conveyor at a constant rate of 1 kg/h. The bed temperature was maintained at approximately 950 °C, while the freeboard temperature reached 650 °C. Bed fluidization was achieved by 6 N m^3^/h of air, which corresponds to an excess air ratio of approximately 1.43, based on an estimated stoichiometric air requirement of 4.2 N m^3^/h for complete combustion of 1 kg of CL. As the air was supplied for in-bed fluidization and no additional air was introduced elsewhere, the excess air ratio in the bed is the same as the overall excess air ratio. The combustion process continued for 4 h each day. At the end of each experiment, the fuel supply and electrical heating were turned off, and the boiler was cooled by continuing the air supply. During the reactor cooling, the air supply was reduced to 3 N m^3^/h to limit the contact between the bed particles and prevent attrition of the bed particle layers. Once the reactor reached room temperature, the air supply was stopped, and bed particle samples were collected directly from the bed. This procedure was repeated over the 4 days of the campaign, resulting in the collection of four bed solid samples with residence times ranging from 4 to 16 h.

### 2.4 Characterization Methods

#### 2.4.1 SEM/EDS Analysis

To investigate the distribution and thickness of the bed particle layers and the structural variations of the bed particles throughout the process, surface and cross sections of both fresh bed particles and samples taken at various time intervals during the campaign were examined using a scanning electron microscope (SEM). The SEM apparatus was a JSM-IT300 (JEOL, Japan) equipped with a backscattered electron (BSE) detector and operated in low-vacuum mode (100 Pa). Elemental analysis of the bed particle layers and different internal features of the bed particles was conducted via energy-dispersive X-ray spectroscopy (EDS) using an X-Max 80 (Oxford Instruments, UK).

To reveal the cross sections of the bed particles and individual bed ash particles (i.e., ash particles derived from the conversion of fuel that remained within the bed material), samples taken after different operation intervals were mounted separately in epoxy resin, allowed to dry for 16 h, and then dry-polished. From each sample, 10 bed particles and 5 bed ash particles were analyzed. Only the largest cross sections from each analyzed bed particle were considered to ensure that the sections were taken from the middle of the bed particles. For each bed sample, 15–20 EDS spot analyses were performed on each bed particle for each feature (inner layer, outer layer, porous regions, rigid bed core). An equivalent number of analyses were also performed on each bed ash particle to ensure a comprehensive compositional assessment. The results were averaged to determine the average elemental composition of each feature. To minimize errors associated with spatial resolution, EDS spots were selected at the center of the bed particle layers. Additionally, EDS intensity mappings for each specific element of interest were conducted over the entire cross-section to determine the compositional distribution throughout both bed and bed ash particles.

An image processing method was used to eliminate human error in measuring the bed particle layer thickness and increase the number of measurement points around each cross-section. This automated process detects the bed particle layer and measures the bed particle thickness at 300–400 points per cross-section, resulting in a total of 3000–4000 measured points for each bed sample. Details on the employed image processing algorithm can be found elsewhere.^50^

#### 2.4.2 XRD Analysis

Individual bed ash particles with sizes below 50 μm were sieved from the oldest bed material samples. The sieved bed ash samples were also analyzed with X-ray diffraction (XRD). XRD analysis was performed using a PANalytical Empyrean diffractometer (Malvern Panalytical, UK), equipped with Cu Kα radiation and a Pixel3D array detector. Qualitative analysis of the diffractograms was performed using PANalytical HighScore Plus 4.9 software in conjunction with the ICDD PDF-5+ 2024 database.

## 3 Results and Discussion

Figures 1 and 2 present backscattered surface SEM images of the bed sample taken from the boiler after 4 and 8 h, respectively. These images reveal ilmenite bed particles alongside individual bed ash particles. These bed ash particles contain a significant amount of undigested Ca-rich particles, likely consisting of lime. The attrition of bed ash particles after 8 h is clearly visible in Figure 2b where the bed ash particles could be seen as comparably finer particles covering ilmenite bed particles. A cross-section of a typical bed ash particle, observed in the sample taken after 8 h, is shown in Figure 2c. The bed ash particles appear relatively homogeneous, sintered, and seem to have undergone partial melting.

**Figure 1:**
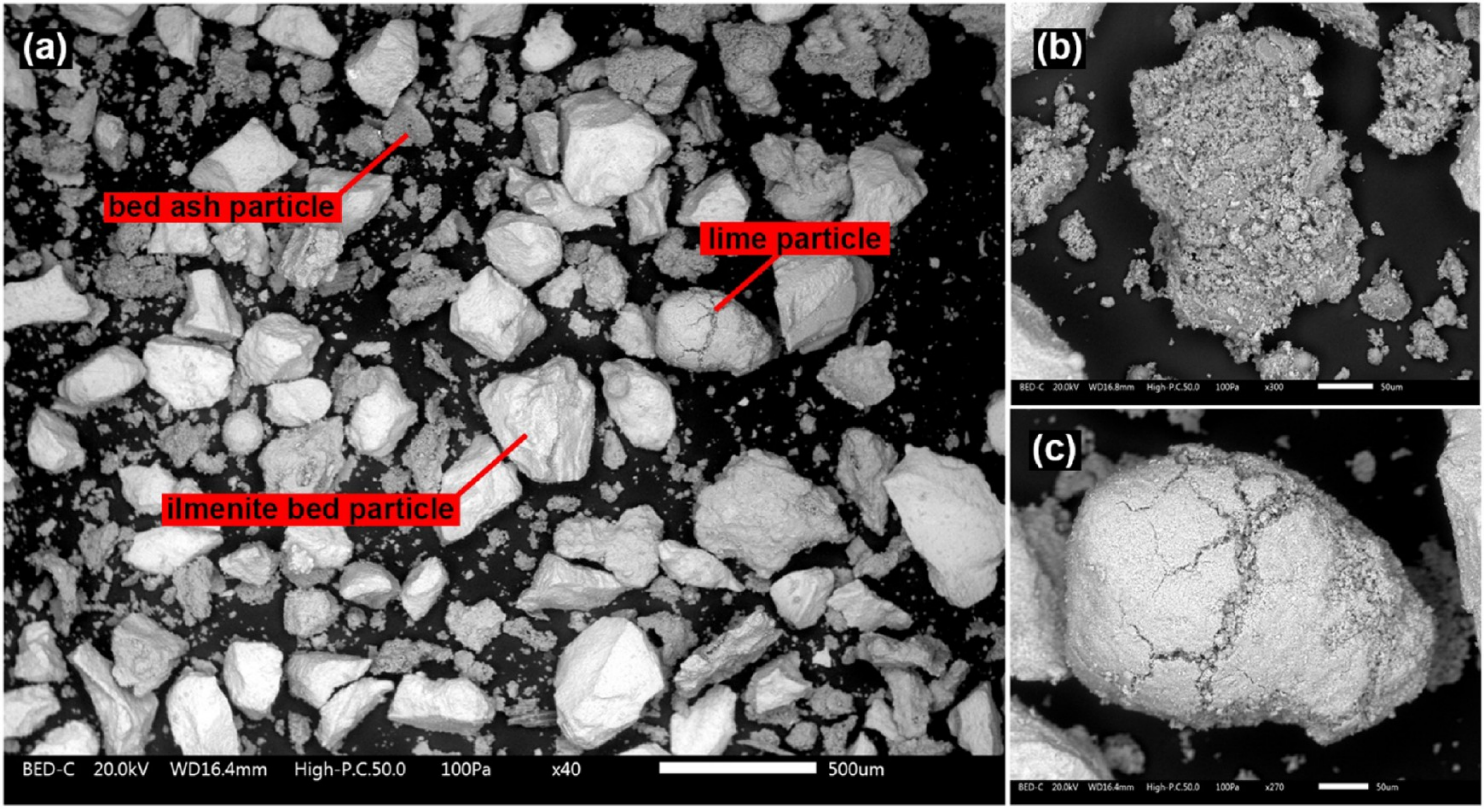
(a) BSE-SEM image of the bed sample taken after 4 h from the BFB_5_, (b) magnified image of an individual bed ash particle, and (c) magnified image of a lime particle found in the same sample.

**Figure 2:**
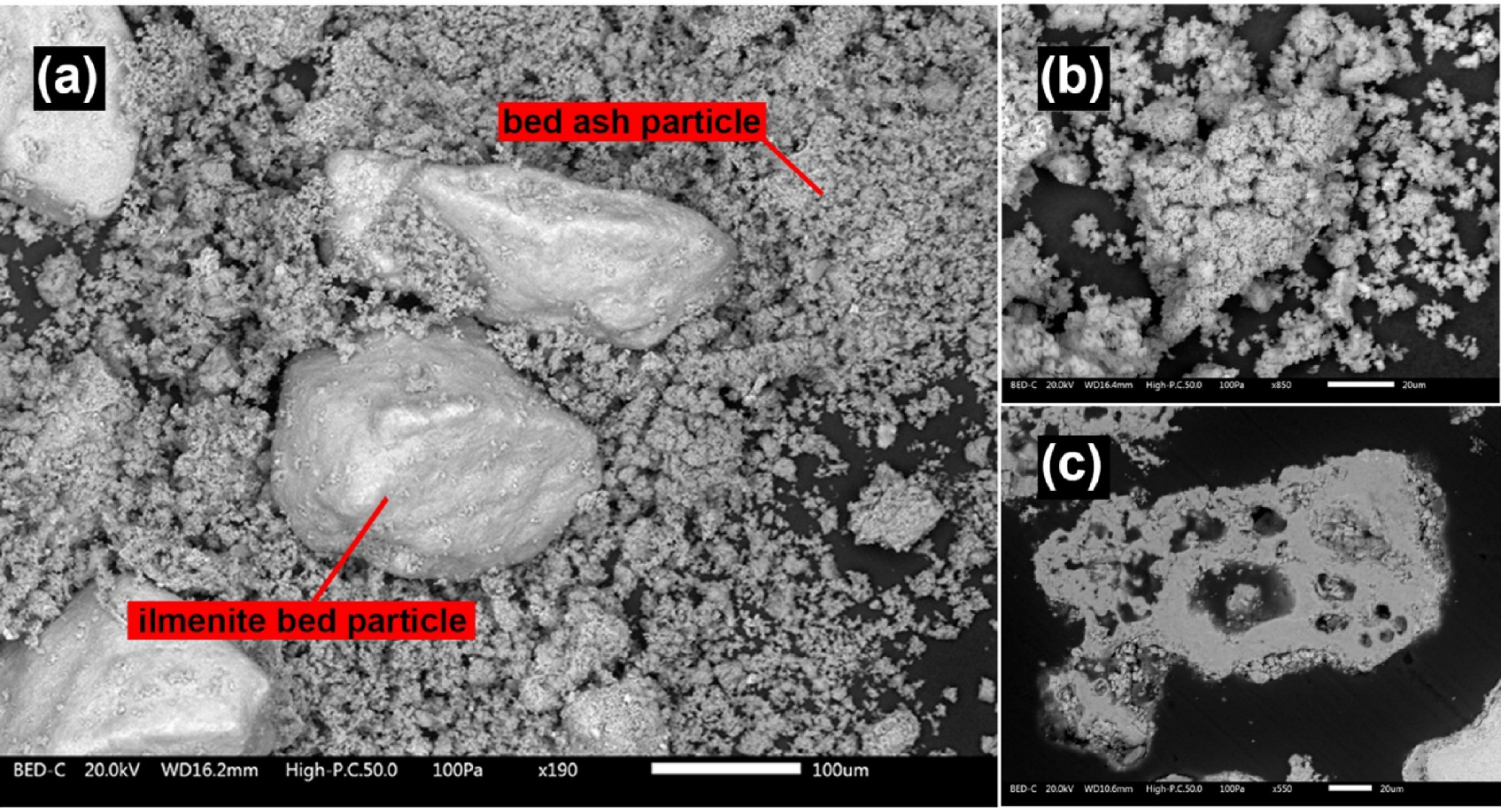
(a) BSE-SEM image of the bed sample taken after 8 h from the BFB_5_, (b) magnified image of individual bed ash particles, , (c) cross-section of a typical individual bed ash particle.

Figure 3 shows cross-sectional SEM images of typical ilmenite bed particles collected from the fluidized bed at different intervals. Even after 4 h from start-up, the formation of a bed particle layer is visible on the bed particles as brighter areas. Beneath this brighter region, a porous region begins to develop after 8 h and continues to grow over time.

**Figure 3:**
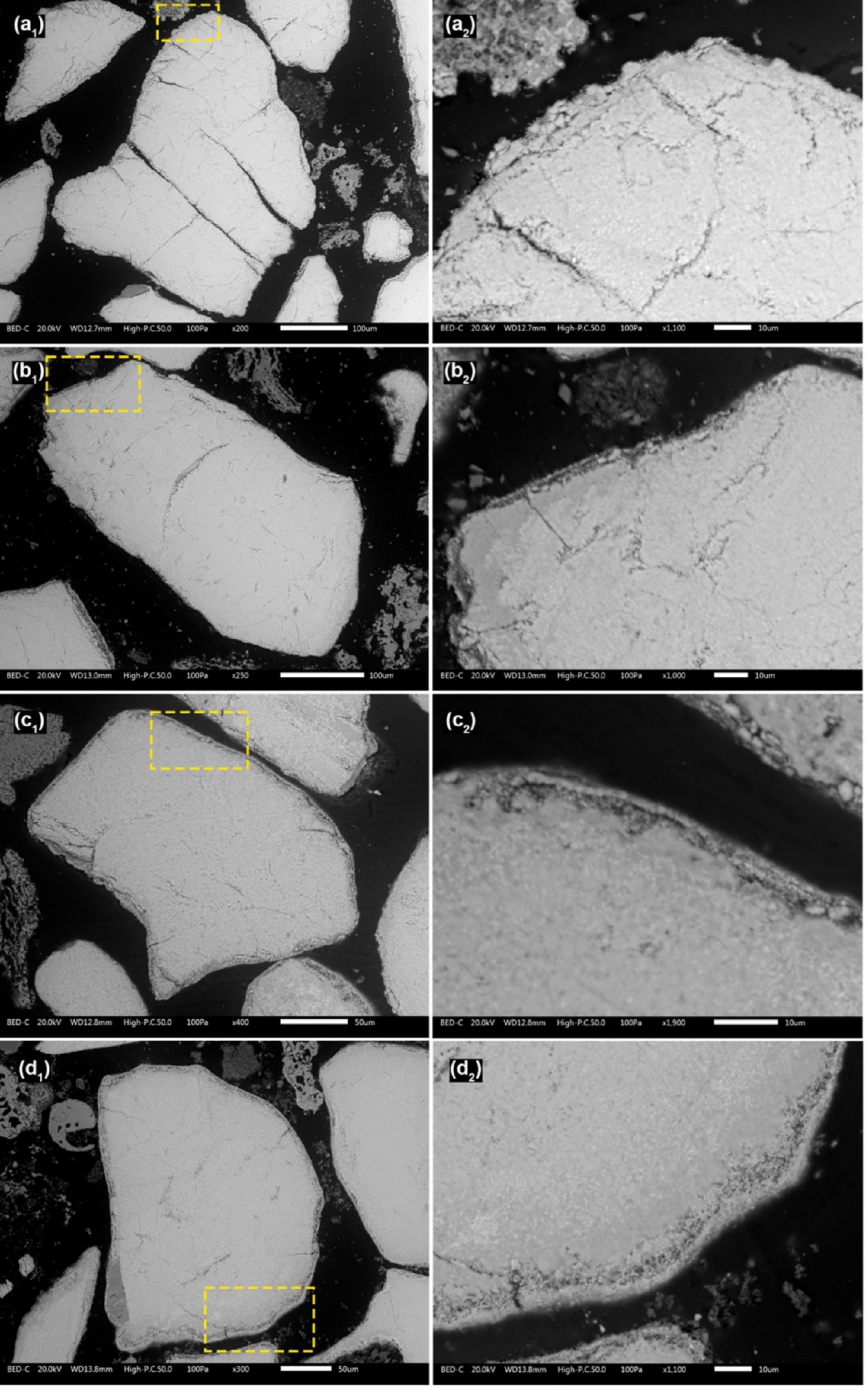
Cross-sectional BSE-SEM images of typical ilmenite bed particles collected from the BFB_5_ after (a) 4 h, (b) 8 h, (c) 12 h, and (d) 16 h after the start-up. The area marked with the yellow dashed lines in (1) is magnified in (2).

To understand the bed particle layer formation process and its characteristics, it is useful to examine the cross-sectional SEM images of the bed particle layer together with the EDS elemental mapping of the main ash-forming elements, as shown in Figures 4–7. This combined approach not only reveals morphological features but also enhances the reliability of distinguishing between the inner and outer layers by correlating structural observations with compositional gradients. Additionally, to compare the abundance of the main ash-forming elements in the bed particle layer, the average elemental composition of the inner and the outer layers at different time intervals is presented in Figure 8. By combining the information from Figures 3 and 4, it can be observed that a Ca-rich inner layer was formed on the surface of the bed particles after 4 h. In addition, an Fe-rich outer layer was also present, as anticipated, due to the tendency of Fe in the ilmenite structure to migrate toward the bed particle surface, where the partial pressure of oxygen is higher. However, despite the presence of the Ca-rich inner layer, the outer Fe-rich layer did not cover the bed particle uniformly and was instead sporadically distributed across the surface.

**Figure 4:**
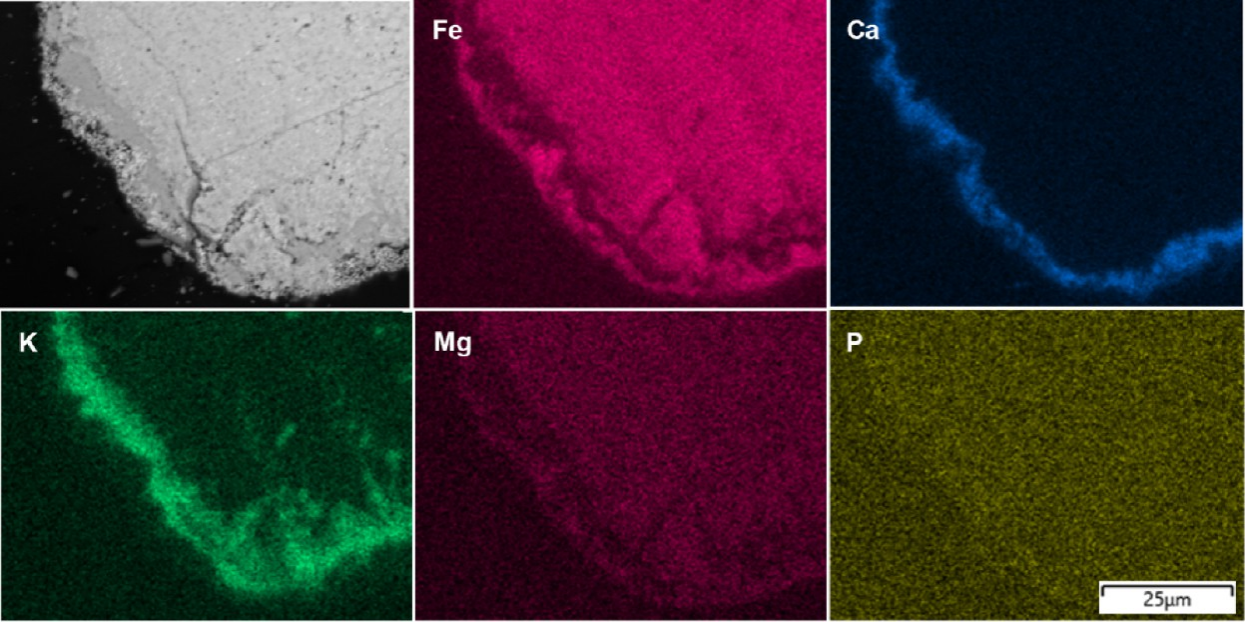
Cross-sectional BSE-SEM image of the bed particles formed after 4 h in a typical ilmenite bed particle collected from the BFB_5_ together with the EDS elemental mapping.

After 8 h (Figure 5), the outer layer covered almost the entire surface of the bed particle and was primarily composed of Ca, P, Mg, and K. This outer layer appeared to be sintered and differed from the heterogeneous outer layer typically observed when woody biomass fuels are used. Fe and Ti were also detected in the outer layer. The presence of Fe is likely due to its continued migration toward the surface. Additionally, because the layer was very thin, the detection of Fe and Ti may have been influenced by signal interference during EDS analysis, where signals from the underlying layers could be captured.

**Figure 5:**
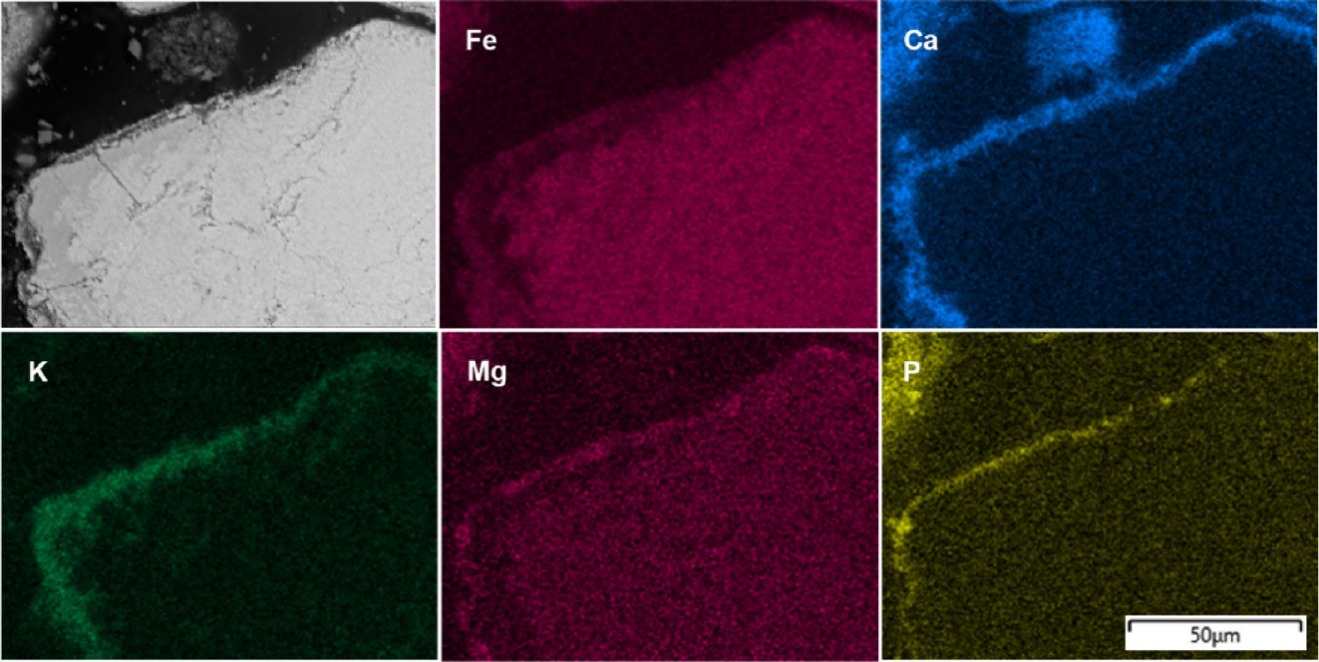
Cross-sectional BSE-EM image of the bed particles formed after 8 h in a typical ilmenite bed particle collected from the BFB_5_, together with the EDS elemental mapping.

In the bed particle samples collected after 12 and 16 h (Figures 6 and 7), the inner layer was observed to be separated into a Ca-rich inner zone and a porous Fe-rich outer zone. As a result, the EDS mappings revealed two distinct Ca-rich regions: one associated with the inner layer and the other with the outer layer, where it was combined with Mg, K, and P, separated by the porous Fe-rich zone.

**Figure 6:**
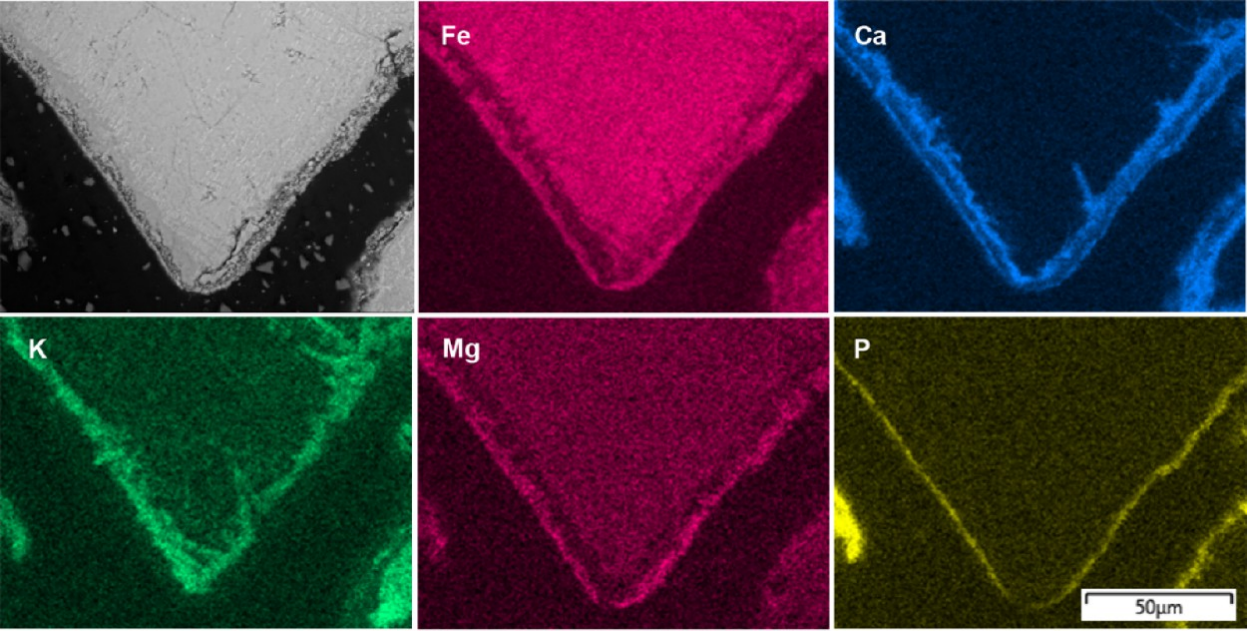
Cross-sectional BSE-SEM image of the bed particles formed after 12 h in a typical ilmenite bed particle collected from the BFB_5_, together with the EDS elemental mapping.

**Figure 7:**
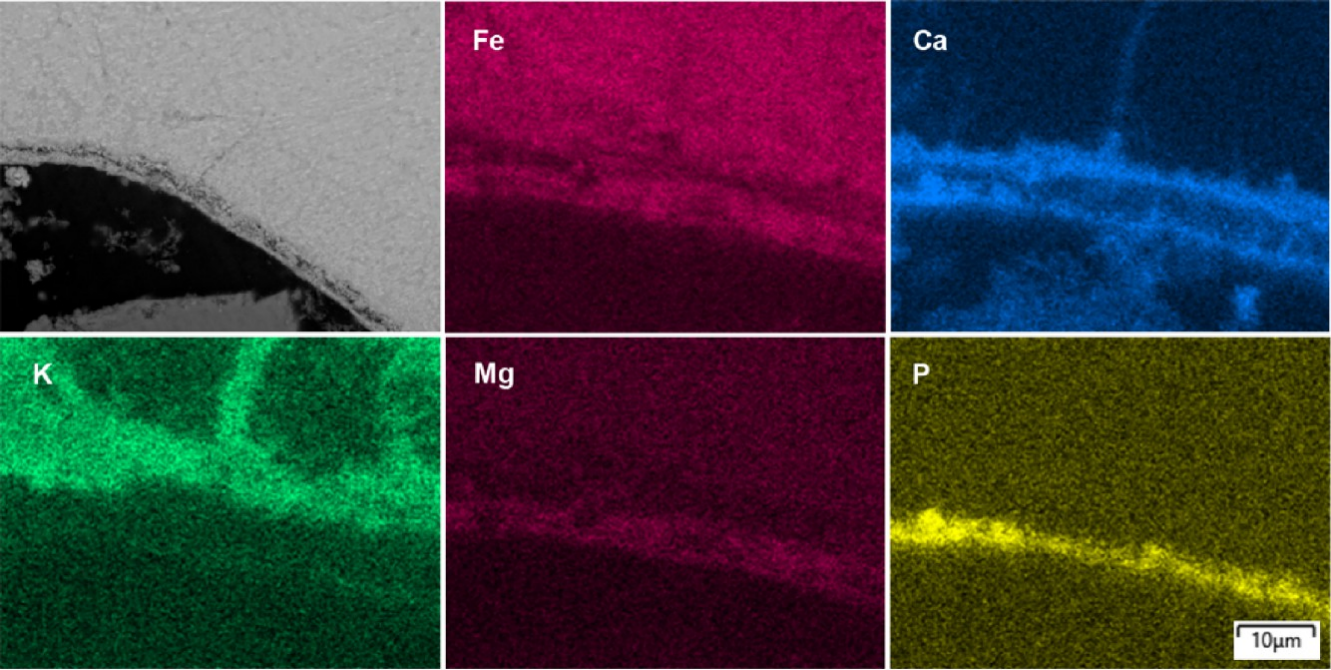
Cross-sectional BSE-SEM image of the bed particles formed after 16 h in a typical ilmenite bed particle collected from the BFB_5_, together with the EDS elemental mapping.

Figure 8 illustrates the average composition of the inner and outer layers at various time intervals throughout the experimental campaign. Over time, an increase in Ca concentration is observed in the inner layer, attributed to Ca diffusion from Ca-rich particles into the core of the bed particle, leading to the formation of Ca-titanate. This trend aligns with findings from previous studies where woody biomass was used as fuel.^20^ However, from the figure, it can be observed that the Ca-titanate is unsaturated. This is due to the fact that the process is diffusion-controlled and, therefore, very slow. Consequently, the duration of the experiment was insufficient for Ca and Ti to reach their equimolar ratio, corresponding to full saturation. Additionally, as the outer layer forms, it limits the direct access of Ca-rich ash particles to the bed particle surface, thereby reducing its subsequent diffusion into the bed particle core.

**Figure 8:**
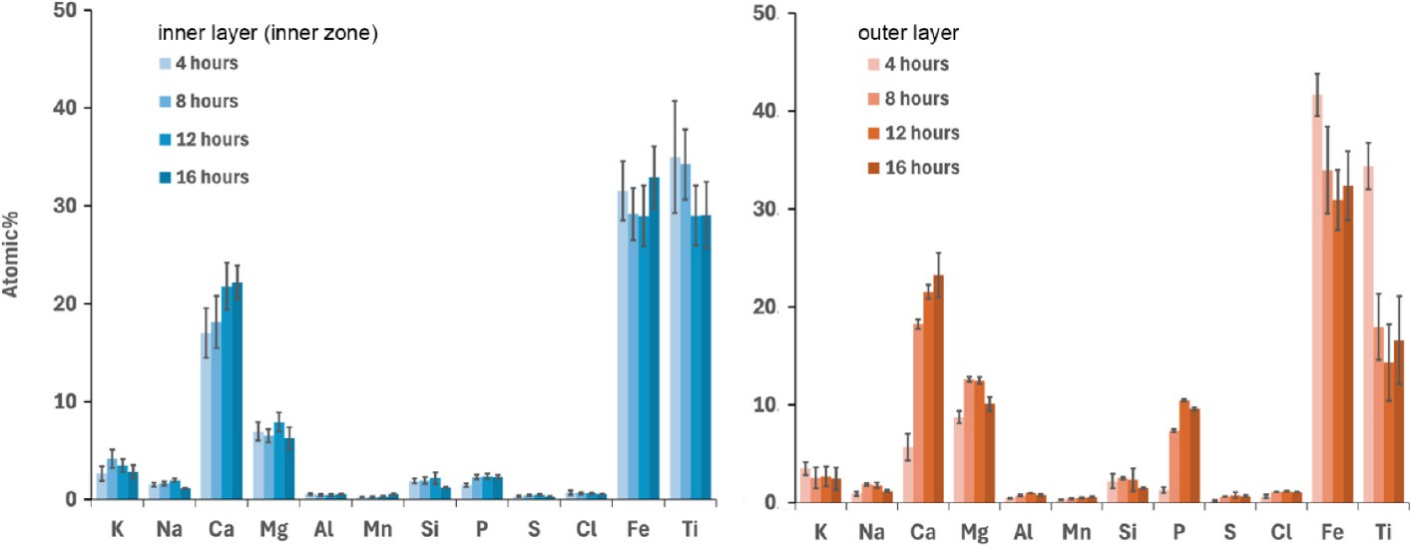
Average concentration of the main ash-forming elements together with Fe and Ti (on a C–O-free basis) in the inner layer and the outer layer formed on ilmenite bed particles taken at different time intervals from the BFB_5_. The error bars show 95% confidence interval for the measured data.

In the outer layer, a noticeable increase in Ca and P concentrations occurs after 4 h, when partially molten ash particles begin to adhere to the inner layer, contributing to the formation of the outer layer.

Figure 9 compares the average elemental composition of the individual bed ash particles with that of the outer layer. To minimize potential signal interference from Fe and Ti in the underlying layers, these two elements are excluded from the figure. It is evident that the composition of the outer layer closely resembles that of the bed ash particles, consistent with previous findings that suggest the outer layer forms from the deposition of fine ash particles onto the surface of the bed particles.^51^ The Fe detected in bed ash particles is likely a result of the attrition of bed particles, which is subsequently incorporated into the ash. The compositional data from the fuel ash analysis suggests that the relative abundance of alkali and alkaline earth elements supports the stoichiometry for orthophosphate formation, which generally reduces the ash melting tendency.^52^ However, as mentioned earlier, the main source of Ca in this case originates from the calcium supplement added to the chickens’ diet. Excess alkali and alkaline earth elements in the ash may interact directly with the bed material. XRD analysis revealed that 52 wt % of the bed ash particles consisted of amorphous material. The crystalline phase was primarily composed of Ca­(OH)_2_, along with smaller amounts of Fe_2_O_3_, CaCO_3_, CaFe_2_O_4_, SiO_2_, and MgO. The only P-containing crystalline phase detected was Ca_5_(PO_4_)_3_OH, accounting for 3 wt % of the total crystalline fraction. This indicates that the majority of P was retained within the amorphous phase of the ash particles. However, the high Ca:P ratio in the fuel ash, supported by the calcium supplements, reduces the likelihood of forming K- and Mg-phosphates, which have lower melting temperatures compared to Ca-phosphates and therefore can significantly increase the risk of bed agglomeration. On the other hand, if the Ca support in the ash were lower, a greater proportion of low-melting compounds would likely form, resulting in more molten ash during combustion. This molten phase tends to be more adhesive, promoting the deposition of ash onto the surface of bed particles and leading to the development of a thicker outer layer. A thicker and more viscous layer can act as a barrier to inward and outward diffusion, which in turn may hinder the outward migration of Fe from the bed material core. Thus, the calcium content in the ash not only influences melting behavior and agglomeration tendency but also indirectly affects the dynamics of layer formation and elemental transport at the particle surface.

**Figure 9:**
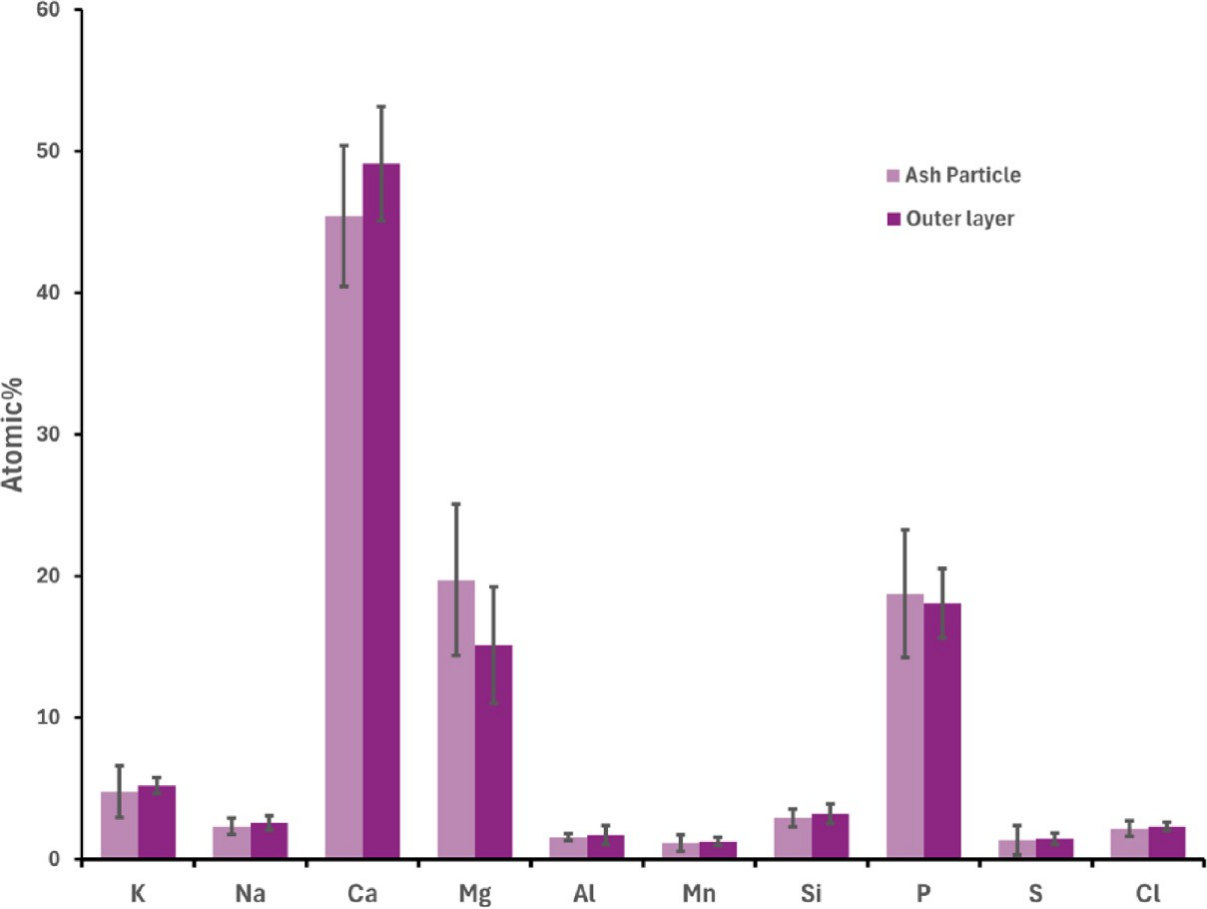
Average concentration of the main ash-forming elements (on a C–O–Fe-Ti-free basis) in the individual bed ash particles and the outer layer for the bed samples taken after 8 h from the BFB_5_. The error bars show 95% confidence interval for the measured data.

In all bed particle samples, a high concentration of K was detected beneath the inner layer, likely due to the formation of K-titanate, as reported in previous studies [30]. The deeper penetration of K into the bed particle core compared to Ca can be attributed to the higher diffusivity of gaseous alkali relative to solid Ca-rich particles.^20,30,53^ As the bed particle ages through the process, gaseous alkali continues to penetrate the core with increasing concentration, as shown in the EDS elemental mapping of K at different intervals from start-up (see Figure 10).

**Figure 10:**
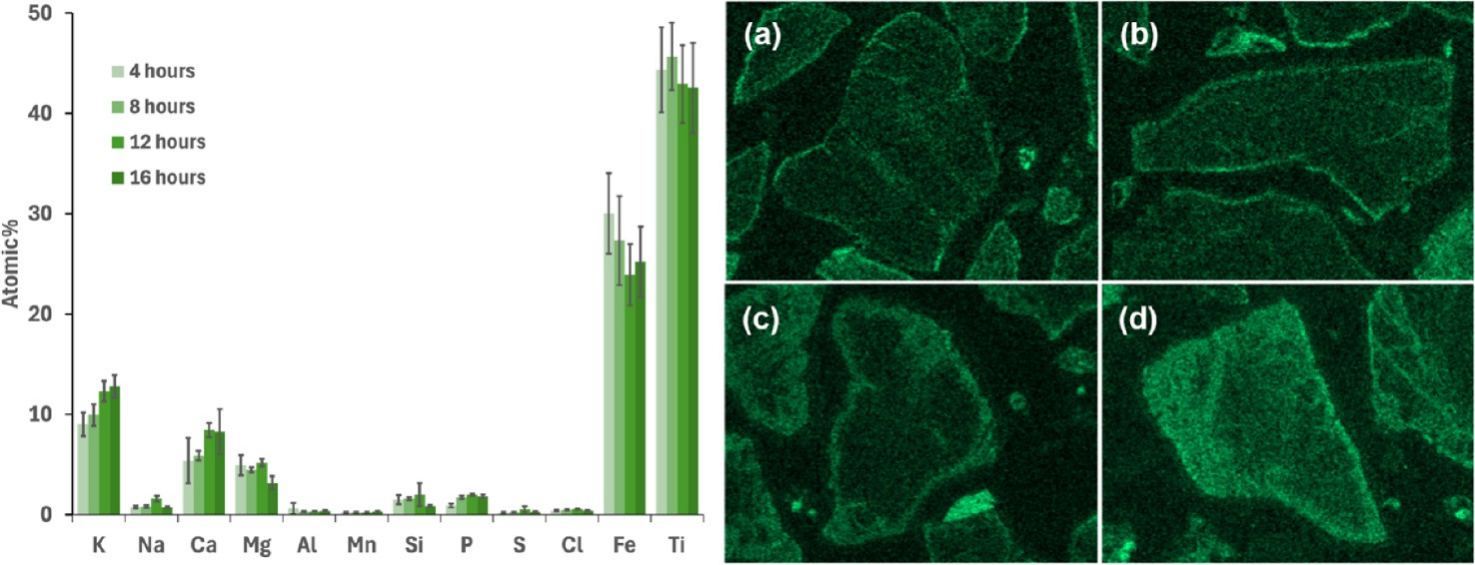
Average concentration of the main ash-forming elements together with Fe and Ti (on a C–O-free basis) in the K-rich areas together with the EDS elemental mapping of K in typical ilmenite bed particles collected from the BFB5 after (a) 4 h, (b) 8 h, (c) 12 h, and (d) 16 h from the start-up. The error bars show 95% confidence interval for the measured data.

The average thickness of both the outer layer and the Ca-rich inner layer was measured and averaged for 10 representative bed particles from each sample (Figure 11). Due to the low phase contrast between the inner layer and the bed particle core, EDS elemental mapping of Ca was employed to delineate the boundaries of the Ca-rich inner layer, allowing for an estimation of bed particle layer thickness based on these boundaries. The variation of measured values around the average, represented as error bars, clearly indicates that the outer layer is uniformly distributed on the bed particle surface, irrespective of surface morphology differences. In contrast, the high variability in measurements around the average for the Ca-rich inner layer suggests that its thickness differs across various morphologies on the bed particle surface.

**Figure 11:**
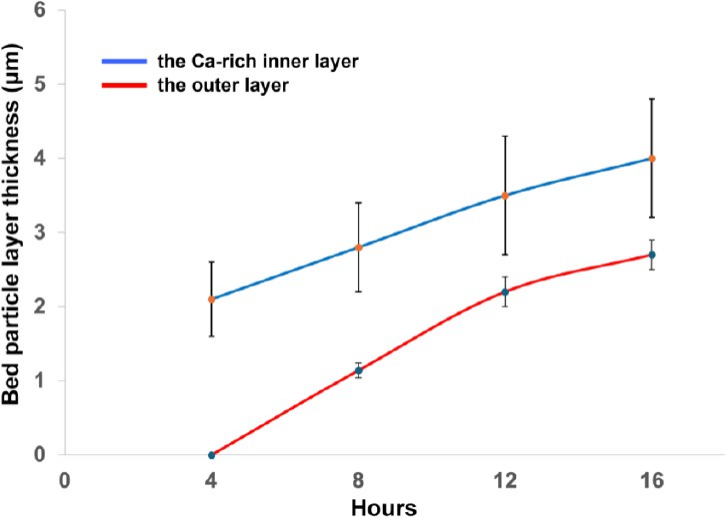
Average thickness of the outer layer (red) and the Ca-rich inner layer (blue) formed on typical ilmenite bed particles collected at different time intervals from the start-up. The error bars show 95% confidence interval for the measured data.

A schematic representation of the bed particle layer formation process for ilmenite bed particles used in the fluidized bed combustion of CL is shown in Figure 12, based on the results above. The initial stage of bed particle layer formation on ilmenite particles in this process appears similar to observations made with woody biomass (Figure 12a).^20^ Throughout the combustion process, bed ash and excreted Ca particles experience attrition, generating abundant Ca-rich particles with sizes below 10 μm (Figure 2). As previously discussed, these Ca-rich particles can adhere to the bed particle surface, supplying Ca that reacts with the bed material to form Ca–Ti-rich layers, likely Ca-titanate. This reaction predominantly occurs on convex areas of the bed particle surface. Simultaneously, the outward migration of Fe from the bed particle core initiates the formation of an outer layer on the surface.^27,54^

**Figure 12:**
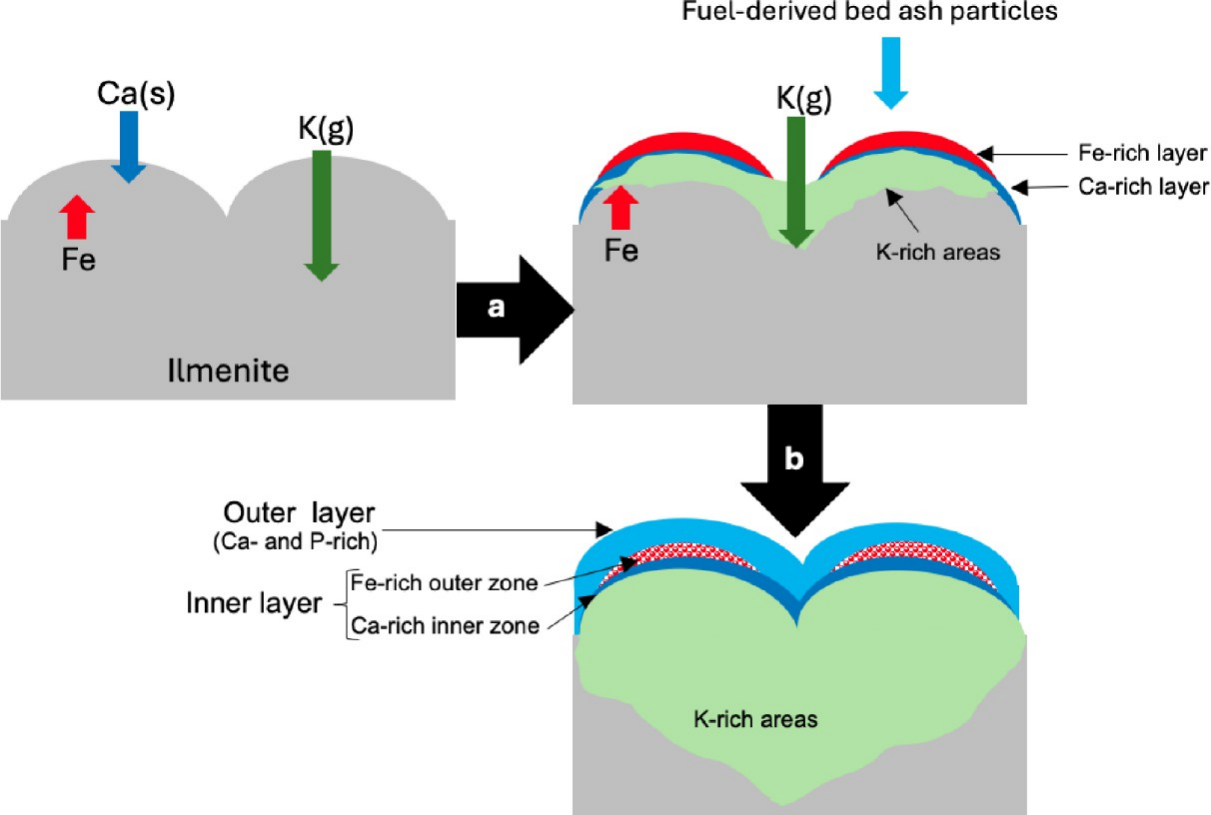
Schematic representation of the bed particle layer formation for ilmenite bed particles utilized in fluidized bed combustion of CL: (a) initial stage after 4 h, (b) after 8 h.

Additionally, gaseous alkali, which has higher mass diffusivity than Ca, can penetrate deeper into the bed particle core and form K-titanate.^27,54^ However, after 8 h, deposition of the fuel-derived bed ash particles begins to control the bed particle layer formation process (Figure 12b). This shift is also evident from the considerable changes in the concentrations of Ca, K, P, and Fe in the outer layer after 8 h (Figure 8). Considering the similar composition of the outer layer to that of the individual bed ash particles (Figure 9), it could be concluded that at this stage, the outer layer is formed due to the deposition of fuel-derived bed ash particles on the bed particle surface. These partially molten bed ash particles develop a sintered outer layer; therefore, despite the heterogeneous outer layer observed in the cases where woody biomass was used as the fuel, the outer layer observed in this study appeared to be homogeneous. Furthermore, it was observed that unlike the layer formation process observed in fluidized bed combustion of woody biomass, the distribution of the outer layer here is identical across different morphologies on the bed particle surface. Therefore, the outer layer uniformly covers the entire bed particle surface. As shown in Figure 8, after 8 h, the inner layer has not reached the saturated equimolar Ca/Ti ratio. This suggests the potential for additional Ca diffusion into the bed particle, which could further develop the inner layer and enhance its ability to inhibit the outward migration of Fe.^20^ However, the uniformly distributed outer layer can inhibit further migration of Fe toward the surface. As a result, Fe becomes trapped under the outer layer, splitting the inner layer into an Fe-rich outer zone (with an average Fe concentration 3–5 atomic% higher than that of the outer layer) and a Ca-rich inner zone. This separation is also observable in Figures 5 and 6, where two Ca-rich layers can be identified in the EDS elemental mappings: one from the Ca–Ti-rich inner layer and the other from the Ca–P–Mg-K-rich outer layer.

Based on the findings of this study, the homogeneous outer layer that forms on the bed particles, covering both convex and concave areas, prevents the outward migration of Fe. As a result, only a thin, porous zone is observed in ilmenite bed particles used with CL, which forms during the initial stage of the process before the outer layer has fully developed. However, gaseous K can still penetrate into the bed particle core through the cracks which are mainly connected to the concaves on the bed particle surface. In contrast, when woody biomass is used, Fe can migrate to the surface through concave areas where the outer layer is absent, allowing the development of porous regions to persist throughout the process. Consequently, under identical conditions, it is expected that ilmenite bed particles used in fluidized bed combustion of woody biomass will exhibit a significantly higher volume fraction of porous areas compared to those used with CL.

In a previous study on ilmenite bed particles using a tubular furnace with synthetic ash, it was shown that in the presence of KH_2_PO_4_, a dense KPO_3_ layer forms on the surface of bed particles. This layer impedes oxygen transport, thereby reducing ilmenite’s reactivity and its overall effectiveness as an oxygen carrier in CLC.^49^ Similarly, the oxygen-carrying capacity of ilmenite bed particles is theoretically expected to be compromised when CL is used, due to the limited transport of Fe to the bed particle surface.^20^ However, a detailed study of the oxygen-carrying capacity of ilmenite bed particles in fluidized bed combustion of CL, in future research, would provide experimental evidence to validate this expectation.

## 4 Conclusions

Bed particle layer formation and characteristics for ilmenite bed particles utilized in fluidized bed combustion of CL were studied in this work. Based on the findings, the following conclusions can be drawn:

- In the initial stage of the process, the bed particle layer formation is governed by the reaction of the bed material with fine fuel-derived Ca-rich particles formed via attrition. The inner layer is developed at this stage and is mainly composed of Ca and Ti.
- After a few hours of operation, the outer layer develops through the direct adhesion of partially molten individual fuel-derived bed ash particles containing Ca, P, Mg, and K, which subsequently controls the ongoing formation of the bed particle layer.
- The thickness of the outer layer remains consistent across different morphologies on the bed particle surface (i.e., convexes and concaves), ensuring an almost uniform coverage across the entire bed particle.
- Due to the uniform and nearly complete coverage of the bed particle surface, the outward migration of Fe is effectively hindered.
